# *Staphylococcus aureus* Bacteremia in Children of Rural Areas of The Gambia, 2008–2015

**DOI:** 10.3201/eid2504.180935

**Published:** 2019-04

**Authors:** Aderonke Odutola, Christian Bottomley, Syed A. Zaman, Jodi Lindsay, Muhammed Shah, Ilias Hossain, Malick Ndiaye, Chidebere D.I. Osuorah, Yekini Olatunji, Henry Badji, Usman N.A. Ikumapayi, Ahmad Manjang, Rasheed Salaudeen, Lamin Ceesay, Momodou Jasseh, Richard A. Adegbola, Tumani Corrah, Philip C. Hill, Brian M. Greenwood, Grant A. Mackenzie

**Affiliations:** London School of Hygiene and Tropical Medicine, London, UK (A. Odutola, C. Bottomley, S.A. Zaman, B.M. Greenwood, G.A. Mackenzie);; Medical Research Council Unit The Gambia at the London School of Hygiene and Tropical Medicine, Banjul, The Gambia (A. Odutola, S.A. Zaman, M. Shah, I. Hossain, M. Ndiaye, C.D.I. Osuorah, Y. Olatunji, H. Badji, U.N.A. Ikumapayi, A. Manjang, R. Salaudeen, M. Jasseh, R.A. Adegbola, T. Corrah, G.A. Mackenzie);; St. George’s University of London, London (J. Lindsay);; King Fahad Medical City, Riyadh, Saudi Arabia (A. Manjang);; Ministry of Health and Social Welfare, Banjul (L. Ceesay);; University of Otago, Dunedin, New Zealand (P.C. Hill);; Murdoch Children’s Research Institute, Parkville, Victoria, Australia (G.A. Mackenzie)

**Keywords:** *Staphylococcus aureus*, bacteremia, The Gambia, children, infants, fatality, epidemiology, surveillance, neonates, case-fatality ratio, bacteria, pneumonia, sepsis, meningitis/encephalitis, pneumococcal conjugate vaccine, incidence, invasive bacterial disease

## Abstract

*Staphylococcus aureus* bacteremia is a substantial cause of childhood disease and death, but few studies have described its epidemiology in developing countries. Using a population-based surveillance system for pneumonia, sepsis, and meningitis, we estimated *S. aureus* bacteremia incidence and the case-fatality ratio in children <5 years of age in 2 regions in the eastern part of The Gambia during 2008–2015. Among 33,060 children with suspected pneumonia, sepsis, or meningitis, we performed blood culture for 27,851; of 1,130 patients with bacteremia, 198 (17.5%) were positive for *S. aureus*. *S. aureus* bacteremia incidence was 78 (95% CI 67–91) cases/100,000 person-years in children <5 years of age and 2,080 (95% CI 1,621–2,627) cases/100,000 person-years in neonates. Incidence did not change after introduction of the pneumococcal conjugate vaccine. The case-fatality ratio was 14.1% (95% CI 9.6%–19.8%). Interventions are needed to reduce the *S. aureus* bacteremia burden in The Gambia, particularly among neonates.

In 2016, invasive bacterial diseases accounted for one quarter of the 5.6 million childhood deaths worldwide (*1*). Most invasive bacterial diseases occur in sub-Saharan Africa and other low- and middle-income countries (*2*). Deaths caused by these diseases outnumber those caused by malaria among children <5 years of age (*3*). The main bacteria implicated in invasive bacterial diseases has been *Streptococcus pneumoniae* and *Haemophilus influenzae* (*4*). However, after the widespread use of conjugate vaccines against *H. influenzae* type b (Hib) and *S. pneumoniae*, Hib disease has decreased considerably (*5*), and vaccine-serotype pneumococcal disease is declining (*6*). The decreased disease incidence associated with these pathogens has led to *Staphylococcus aureus* becoming a relatively more common cause of invasive bacterial disease (*7*). The clinical spectrum of *S. aureus* disease ranges from life-threatening invasive diseases, such as septicemia, pneumonia, osteomyelitis, endocarditis, meningitis, and brain abscess, to less severe skin and soft tissue infections. *S. aureus* bacteremia is often used as a marker of invasive *S. aureus* disease (*8*).

In high-income countries, *S. aureus* bacteremia is the second most common cause of neonatal sepsis, after group B *Streptococcus* (*9*). From the 1970s through the 2000s, the incidence of *S. aureus* bacteremia among children <16 years of age increased in several countries (*10*), probably because of the increased use of central venous catheters and other factors (*10*). In the 2010s, the incidence of *S. aureus* bacteremia remained stable (*11*) or decreased (*10*).

In Africa, *S. aureus* bacteremia is a common cause of invasive bacterial disease in children. Before the introduction of the Hib vaccine and pneumococcal conjugate vaccine (PCV), population-based studies in Kenya and Mozambique showed that *S. aureus* was the most common gram-positive pathogen among neonates with sepsis (*4*,*12*). Also, hospital-based studies showed *S. aureus* to be the most common cause of invasive bacterial disease in children <3 months of age in The Gambia (*13*) and one of the main causes of invasive bacterial disease in children <5 years of age in Nigeria (*14*).

Few population-based studies have been conducted in sub-Saharan Africa on the incidence of *S. aureus* bacteremia. In South Africa, a population-based study of children <13 years of age in an area with a high HIV prevalence indicated an incidence of 26 cases/100,000 person-years (*15*). A study in Kenya involving children admitted to a secondary healthcare facility showed an incidence of 27 cases/100,000 person-years in children <5 years of age; the highest incidence was in infants (89 cases/100,000 person-years) (*4*). However, variation in the age groups studied and methods used preclude direct comparison of these studies (*4*,*12*,*13*,*15*). After introduction of the Hib vaccine and before the introduction of PCV, a hospital-based study in The Gambia reported that *S. aureus* was the most common cause of bacteremia (*16*).

Given the paucity of population-based data on the epidemiology of *S. aureus* bacteremia in sub-Saharan Africa, we studied the incidence, clinical characteristics, case-fatality rate, and risk factors for *S. aureus* bacteremia in young children in a rural region of The Gambia. We also explored the association of *S. aureus* bacteremia with the introduction of PCV.

## Methods

### Study Site and Population

**Figure 1:**
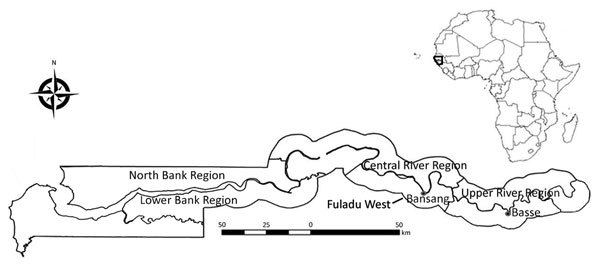
Regions surveilled for *Staphylococcus aureus* bacteremia among children <5 years of age through the Basse and Fuladu West Health and Demographic Surveillance Systems, The Gambia, 2008–2015. Inset indicates location of The Gambia in Africa.

Surveillance for septicemia, pneumonia, and meningitis was performed among children ≥2 months of age residing in Basse in the Upper River Region of The Gambia through the Basse Health and Demographic Surveillance System (BHDSS) (Figure 1). We established the BHDSS in 2007, and the population in this surveillance area (≈179,000 persons in 2015, 19% <5 years of age) is enumerated every 4 months. The BHDSS is served by 5 satellite clinics and the Basse Health Centre (Basse, The Gambia), a primary and secondary healthcare facility with 25 beds to care for children. During 2011–2015, surveillance was extended to include all residents <5 years of age, and a similar surveillance was set up in the adjacent district of Fuladu West for all residents <5 years of age during a similar time range (2012–2014) through the Fuladu West Health and Demographic Surveillance System (FWHDSS; Figure 1). The population in Fuladu West is enumerated annually (population 92,464 in 2014, 18% <5 years of age). The FWHDSS is served by Bansang Hospital (Bansang, The Gambia) and 2 satellite clinics. Every resident in the areas surveilled by the BHDSS and FWHDSS was assigned a unique identifier.

The conjugate vaccine for Hib was introduced into the Gambian National Programme on Immunization in 1997, and the vaccine for pneumococcus was introduced in 2009. The 7-valent PCV (PCV7) was replaced by the 13-valent vaccine (PCV13) in 2011. In 2012, vaccine coverage for the third dose of the diphtheria-pertussis-tetanus vaccine in these regions surveilled was 81.7% (*17*). In The Gambia, transmission of *Plasmodium falciparum* is largely restricted to the short rainy season during July–November (*18*).

### Surveillance Procedures

During May 12, 2008–December 31, 2015, nurses screened all children 2–59 months of age who arrived at a health center participating in the surveillance and who had a unique identifier for septicemia, pneumonia, and meningitis, according to standardized criteria (also referred to as referral surveillance) (*19*). Children who were admitted and children who were treated as outpatients were screened. Children who screened positive were referred to clinicians who used standardized criteria for assessment and investigation (*19*). Data collected included age, sex, anthropometric measurements, signs and symptoms, and suspected diagnosis. Blood was collected for culturing, and depending on clinical presentation, cerebrospinal fluid, lung aspirate, or pleural fluid samples were have also been collected for conventional microbiological tests (*6*). Rapid diagnostic tests for malaria (ICT Malaria *P.f.* Antigen; ICT Diagnostics, http://www.ictdiagnostics.co.za) were routinely performed during the rainy season and at other times at the discretion of the clinician.

During March 1, 2011–December 31, 2015, surveillance was expanded in the BHDSS to include all children 0–59 months of age who were admitted with an acute medical problem from whom a blood sample was collected for culture (also referred to as admission surveillance). During September 12, 2011–December 31, 2014, a similar admission surveillance was conducted for children 0–59 months of age admitted with an acute medical problem using the FWHDSS. All *S. aureus* bacteremia cases were linked to the Health and Demographic Surveillance System databases by using the unique identifier.

### Laboratory Methods

We collected 1–3-mL blood samples from all patients with suspected septicemia, pneumonia, or meningitis; inoculated blood samples into BACTEC bottles (Becton Dickinson, https://www.bd.com); and incubated them in an automated BACTEC 9050 Blood Culture System (Becton Dickinson) for a maximum of 5 days. We subcultured positive cultures on blood agar plates and confirmed isolates as *S. aureus* by using catalase and coagulase tests. We classified cultures that grew *Bacillus* spp., C*orynebacterium* spp., and coagulase-negative *Staphylococcus* as contaminated. We used standard methods to investigate other body fluid samples collected for microbiological tests (*20*). We used disc diffusion methods to determine antimicrobial drug susceptibility according to the Clinical and Laboratory Standards Institute guidelines (*21*). We categorized all *S. aureus* isolates resistant to cefoxitin as methicillin-resistant.

We defined *S. aureus* bacteremia cases as clinically suspected cases of septicemia, pneumonia, meningitis, osteomyelitis, septic arthritis, pyomyositis, or abscess identified by using standardized criteria (*19*) in patients from whom *S. aureus* was isolated from their blood.

### Statistical Methods

We used referral and admission surveillance data for statistical analyses. The unique identifier assigned to every patient enabled us to avoid duplication of data in our data set. We used the referral surveillance data to investigate trends in incidence because these data covered a longer period (2008–2015) than the admissions surveillance data (2011–2015). We used both the admission and referral surveillance data to estimate age-specific incidence and the case-fatality ratio (CFR).

We obtained incidence estimates by dividing the number of *S. aureus* bacteremia cases by the number of person-years at risk using the estimated midyear population. To account for the shorter period of observation in 2008 (May 12–December 31), we calculated person-years at risk as the midyear population multiplied by 234/365. We calculated incidence in neonates using 2 methods, first as cases per 1,000 live births and second as cases per 100,000 person-years. We defined the neonatal period as the time from birth to 28 days of age.

With the referral surveillance data, we assessed trends in incidence over time and variation in incidence before (pre-PCV period, May 12, 2008–May 11, 2010) and after (PCV13 period, January 1, 2013–December 31, 2015) the introduction of PCV7 using Poisson regression with robust error variance to allow for overdispersion. To account for the increased rate of eligible patients requiring blood culture over time, we adjusted the number of *S. aureus* bacteremia cases of each age group and year by multiplying by the ratio of the annual rate of eligible children enrolled over the mean rate of eligible children enrolled during the entire study period (*6*). For the denominators of the pre-PCV and PCV13 periods, we used the average of the corresponding midyear populations indicated by the BHDSS.

We defined CFR as the number of patients with *S. aureus* bacteremia who died before discharge divided by the total number of patients with *S. aureus* bacteremia. We identified potential risk factors for death before discharge using logistic regression, although surveillance was not designed to assess risk factors. We generated weight-for-age and weight-for-height z-scores using the 2006 World Health Organization child growth standards (https://www.who.int/childgrowth/standards/technical_report). We considered children with weight-for-age z-scores <3 SDs from the median weight-for-age as severely underweight and weight-for-height z-scores <3 SDs from the median weight-for-height as severely stunted. We performed analyses using Stata 14.0 (https://www.stata.com/stata14) and considered p values <0.05 as the criterion for statistical significance.

### Ethics Statement

Ethics approval for this study was granted by The Gambia Government/Medical Research Council Joint Ethics Committee and the London School of Hygiene and Tropical Medicine Ethics Committee. We obtained written informed consent from the parents or guardians of all patients.

## Results

**Figure 2:**
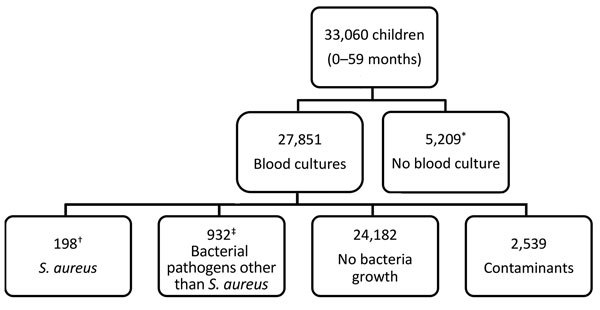
Flowchart of participants included and excluded in study of *Staphylococcus aureus* bacteremia incidence in children <5 years of age, The Gambia, 2008–2015. Participants were identified through the Basse and Fuladu West Health and Demographic Surveillance Systems. In total, 521 cases were identified through referral surveillance and 418 through admission surveillance. *Reasons for not having blood culture done included unsuccessful venipuncture (n = 487), declined consent for venipuncture (n = 416), declined consent to join study (n = 249), and unknown (n = 4,057). †In total, 76 children were identified through referral surveillance and 122 through admission surveillance. ‡Seven patients had polymicrobial bacteremia (*S. aureus* and a second bacterial pathogen).

In total, 33,060 children met the criteria for investigation, and 27,851 (84.2%) blood samples were collected and cultured (Figure 2). Contaminants grew in the cultures of 2,539 (9.1%) blood samples; these samples were excluded from analysis because contamination can mask an *S. aureus* bacteremia diagnosis.

### Bacteremia

Bacteremia was identified in 1,130 children 0–59 months of age (Table 1). *S. aureus* was isolated in 198 (17.5%) children with bacteremia (76 identified through referral surveillance and 122 admission surveillance) and was the most common cause of bacteremia in neonates (46.4%, 84/181). Pathogens other than *S. aureus* were isolated from 932 children: *S. pneumoniae* (35.0%, n = 326), *Salmonella* spp. (15.1%, n = 141), and *Escherichia* spp. (10.7%, n = 100). In 7 children with bacteremia, *S. aureus* and a second bacterial pathogen were isolated.

**Table 1:** Characteristics of patients <5 years of age with suspected pneumonia, septicemia, or meningitis with or without *Staphylococcus aureus* bacteremia identified through 2 surveillance systems, The Gambia, 2008–2015*

Patient characteristic	Patients with *S. aureus* bacteremia, n = 198	Patients with bacteremia caused by other pathogen, n = 932	Patients without bacteremia, n = 24,182
Age, mo			
<1	84/198 (42.4)	97/932 (10.4)	1,911/24,177 (7.9)
1–11	61/198 (30.8)	310/932 (33.3)	8,675/24,177 (35.9)
12–23	33/198 (16.7)	265/932 (28.4)	7,505/24,177 (31.0)
24–59	20/198 (10.1)	260/932 (27.9)	6,086/24,177 (25.2)
Sex			
M	97/198 (49.0)	532/932 (57.1)	13,740/24,177 (56.8)
F	101/198 (51.0)	400/932 (42.9)	10,437/24,177 (43.2)
Severely stunted†	20/109 (18.3)	216/884 (24.4)	3,425/21,736 (15.8)
Mid-upper arm circumference <11 cm	81/198 (40.9)	184/932 (19.7)	3,080/24,182 (12.7)
Admitted in previous 2 weeks	31/162 (19.1)	157/843 (18.6)	3,995/21,897 (18.2)
Hospital stay, d, median (IQR)	5 (2–6)	4 (3–6)	3 (2–4)
Disease onset during wet season‡	97/198 (49.0)	335/932 (35.9)	10,335/24,171 (42.8)
Died	28/198 (14.1)	161/932 (17.3)	860/24,182 (3.6)
Symptoms			
Cough	103/198 (52.0)	675/928 (72.7)	19,523/24,148 (80.8)
Difficult breathing	89/197 (45.2)	535/927 (57.7)	14,280/24,102 (59.2)
Prostration	29/197 (14.7)	147/918 (16.0)	1,602/23,906 (6.7)
Diarrhea	38/190 (20.0)	271/861 (31.5)	5,798/22,772 (25.5)
Convulsion	8/198 (4.0)	72/927 (7.8)	1,174/24,127 (4.9)
Signs			
Lower chest wall in-drawing	164/198 (82.8)	732/927 (79.0)	17,856/24,129 (74.0)
Meningism	1/192 (0.5)	34/867 (3.9)	174/22,841 (0.8)
Altered level of consciousness	124/193 (64.2)	407/873 (46.6)	9,590/23,518 (40.8)
Axillary temperature			
<36.5°C	18/198 (9.1)	79/932 (8.5)	2,405/24,182 (9.9)
36.5°C–37.5°C	40/198 (20.2)	147/932 (15.8)	6,819/24,182 (28.2)
>37.5°C	140/198 (70.7)	706/932 (75.7)	14,958/24,182 (61.9)
Pulse rate, beats/min§			
Increased for age	84/198 (42.4)	621/932 (66.6)	15,107/24,182 (62.5)
Respiratory rate, breaths/min¶			
Increased for age	128/198 (64.6)	682/932 (73.2)	17,157/24,177 (71.0)
Oxygen saturation <92%	33/198 (16.7)	116/932 (12.4)	2,140/24,182 (8.8)
Suspected diagnosis#			
Septicemia	109/194 (56.2)	434/896 (48.4)	8,549/23,068 (37.1)
Pneumonia	55/194 (28.4)	347/896 (38.8)	13,244/23,068 (57.4)
Meningitis	13/194 (6.7)	96/896 (10.7)	718/23,068 (3.1)
Other focal sepsis	17/194 (8.8)	19/896 (2.1)	557/23,068 (2.4)
Malaria positivity**	14/131 (10.7)	84/723 (11.6)	3,276/21,626 (15.1)

*Values are no. patients/total no. in category (%) except as indicated. Surveillance data are from the Basse Health and Demographic Surveillance System and the Fuladu West Health and Demographic Surveillance System. IQR, interquartile range.  †Defined as weight-for-height z-score <3 SDs from median weight-for-height for the corresponding age group. We calculated weight-for-height using z-scores from the 2006 World Health Organization child growth standards in Stata 14.0 (https://www.stata.com/stata14). Neonates were not included in weight-for-height measurements. ‡The wet season occurs during July–November and the dry season during December–June. §The reference ranges for pulse rates were 70–190 beats/min for children <1 month of age, 80–160 beats/min for children 1–11 months of age, 80–130 beats/min for children 1–2 years of age, 80–120 beats/min for children 3–4 years of age, 75–115 beats/min for children 5–6 years of age, 70–110 beats/min for children 7–9 years of age, and 60–100 beats/min for children >10 years of age. ¶Increased respiratory rate was defined as >60 breaths/min for children <2 months of age, >50 breaths/min for children 2–12 months of age, >40 breaths/ min for children >1–5 years of age. #Surveillance diagnosis was categorized into mutually exclusive groups in order of severity; meningitis was considered more severe than septicemia, which was considered more severe than pneumonia. **Malaria was tested using a rapid diagnostic test (ICT Malaria *P.f.* Antigen, ICT Diagnostics, http://www.ictdiagnostics.co.za).

### Patient Characteristics

Using the combined admission and referral surveillance data, we found that 18.2% (4,541/24,885) of all patients were severely underweight and 10.9% (2,658/24,405) were severely stunted; 18.3% (4,183/22,902) of patients were admitted in the 2 weeks before disease onset. Antimicrobial drug use in the week before onset of signs and symptoms was uncommon. Most patients had fever (≥37.5°C) and tachypnea (Table 1).

Among patients with *S. aureus* bacteremia, a diagnosis of suspected septicemia was made in 56.2%, suspected pneumonia in 28.4%, and suspected meningitis in 6.7%. The median duration of hospital stay was 5 (interquartile range 2–6) days (Table 1).

Cough and difficult breathing were experienced more often by patients without bacteremia or with bacteremia caused by other pathogens than by patients with *S. aureus* bacteremia (Table 1). *S. aureus* bacteremia patients were more likely to have a diagnosis of suspected septicemia or other focal sepsis and less likely to have a diagnosis of suspected pneumonia than patients without bacteremia or with bacteremia caused by other pathogens (Table 1).

### Incidence and Risk Factors for *S. aureus* Bacteremia

Using the combined referral and admission surveillance data (2011–2015 in BDHSS and 2012–2014 in FWDHSS), we found the incidence of *S. aureus* bacteremia to be 78 (95% CI 67–91) cases/100,000 person-years in children 0–59 months of age. The incidence was highest among neonates (2,080 [95% CI 1,621–2,627] cases/100,000 person-years, 3.5 [95% CI 2.9–4.7] cases/1,000 live births) and decreased in older age groups (Table 2). Incidence of *S. aureus* bacteremia in the 1–11-month age group was 133 (95% CI 99–174) cases/100,000 person-years, and incidence in the 1–4-year age group was 27 (95% CI 20–36) cases/100,000 person-years. Among the 84 *S. aureus* bacteremia cases in neonates, 13 (15.5%) presented in the first week of life and 35 (41.7%) in the second. The incidence of *S. aureus* bacteremia was higher in the wet season than in the dry season (Table 2).

**Table 2:** Factors associated with *Staphylococcus aureus* bacteremia in children <5 years of age identified through 2 surveillance systems, The Gambia, 2011–2015*

Variable	No. cases/no. person-years at risk	Incidence, cases/100,000 person-years	Incidence rate ratio (95% CI)	p value
Age, mo				
24–59	18/128,994	14.0	1	
12–23	29/44,433	65.3	4.70 (2.6–8.4)	
1–11	53/39,969	132.6	9.50 (5.6–16.2)	
<1	70/3,367	2079.0	148.99 (88.8–250.1)	<0.001
Sex				
M	82/107,515	76.3	1	
F	88/109,248	80.6	1.06 (0.8–1.4)	0.72
Season				
Dry	85/144,508	58.8	1	
Wet	85/72,255	117.6	2.00 (1.5–2.7)	<0.001

*Surveillance data are from the Basse Health and Demographic Surveillance System and the Fuladu West Health and Demographic Surveillance System

### Trends in Incidence of *S. aureus* Bacteremia

**Figure 3:**
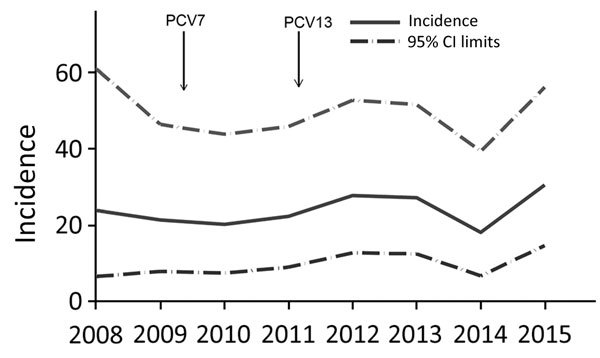
Unadjusted annual incidence of *Staphylococcus aureus* bacteremia (cases/100,000 person-years) in children 2–59 months of age, Basse, The Gambia, 2008–2015. Cases were identified by referral surveillance through the Basse Health and Demographic Surveillance System. Arrows indicate introduction of PCV7 and PCV13. PCV7, 7-valent pneumococcal conjugate vaccine; PCV13, 13-valent pneumococcal conjugate vaccine.

Using referral surveillance data (2008–2015 in BDHSS), we found the mean annual incidence of *S. aureus* bacteremia in children 2–59 months of age to be 22.3 (95% CI 16.7–29.2) cases/100,000 person-years. The incidence did not change over this period (p value for trend = 0.28), although PCV vaccination coverage increased during this period (Figure 3).

Using the referral surveillance data, we observed that 9 cases (10 cases after enrollment rate adjustment) of *S. aureus* bacteremia occurred in the pre-PCV period and 26 cases (23 cases after enrollment rate adjustment) in the PCV13 period. The crude *S. aureus* bacteremia incidence was 16 cases/100,000 person-years in the pre-PCV period and 26 cases/100,000 person-years in the PCV13 period (incidence rate ratio 1.6, 95% CI 0.8–3.5; p = 0.19). With the increasing size of the population and after adjusting for increased enrollment of eligible children over time, no significant increase in *S. aureus* bacteremia incidence was found between the pre-PCV (18 cases/100,000 person-years) and the PCV13 (23 cases/100,000 person-years) periods (incidence rate ratio 1.3, 95% CI 0.6–2.7; p = 0.49).

### CFRs and Risk Factors Associated with Fatality

In total, 28 deaths occurred among 198 *S. aureus* bacteremia patients before discharge (CFR 14.1%, 95% CI 9.4%–20.4%) (Table 1). In comparison, the CFR among patients without bacteremia was 3.6% (95% CI 3.3%–3.8%) and among patients with bacteremia with other pathogens 17.2% (95% CI 14.7%–20.1%). The *S. aureus* bacteremia CFR did not vary by year (p value for trend = 0.75) or age group (p value for trend = 0.99). Deaths associated with *S. aureus* bacteremia most often occurred on the day of admission (71.4%, 20/28). During 2011–2015, *S. aureus* bacteremia deaths accounted for 7.0% (12/171) of all deaths in neonates and 3.6% (24/662) of all deaths in children <5 years of age. The risk factors associated with death from *S. aureus* bacteremia were prostration at clinical presentation and musculoskeletal swelling with or without tenderness (Table 3).

**Table 3:** Sociodemographic and clinical parameters associated with death from *Staphylococcus aureus* bacteremia among children <5 years of age identified through 2 surveillance systems, The Gambia, 2008–2015*

Parameter	Deaths/persons at risk (%)	Unadjusted OR (95% CI)	p value	Adjusted OR (95% CI)†	p value
Age, mo					
<1	13/84 (15.5)	Referent		Referent	
1–11	8/61 (13.1)	0.8 (0.3–2.1)		0.9 (0.4–2.6)	
12-23	4/33 (12.1)	0.8 (0.2–2.5)		1.3 (0.4–4.6)	
24-59	3/20 (15.0)	1.0 (0.3–3.8)	0.96‡	1.1 (0.3–4.6)	0.96
Sex					
M	16/97 (16.5)	Referent			
F	12/101 (11.9)	0.7 (0.3–1.5)	0.35		
Severely stunted§					
No	20/150 (13.3)	Referent			
Yes	5/41 (12.2)	0.9 (0.3–2.6)	0.85		
Axillary temperature					
36.5°C–37.5°C	4/18 (22.2)	Referent			
<36.5°C	4/40 (10.0)	0.4 (0.1–1.8)			
>37.5°C	20/140 (14.3)	0.6 (0.2–2.0)	0.48		
Pulse rate, beats/min¶					
Within reference ranges	13/114 (11.4)	Referent			
Increased for age	15/84 (17.9)	1.7 (0.8–3.8)	0.20		
Respiratory rate, breaths/min#					
Within reference ranges	8/70 (11.4)	Referent			
Increased for age	20/128 (15.6)	1.4 (0.6–3.5)	0.41		
Need for oxygen supplementation					
No	21/165 (12.7)	Referent			
Yes	7/33 (21.2)	1.9 (0.7–4.8)	0.22		
Season					
Dry	18/101 (17.8)	Referent			
Wet	10/97 (10.3)	0.5 (0.2–1.2)	0.13		
Cough					
No	13/95 (13.7)	Referent			
Yes	15/103 (14.6)	1.1 (0.5–2.4)	0.86		
Difficult breathing					
No	14/108 (13.0)	Referent			
Yes	14/89 (15.7)	1.3 (0.6–2.8)	0.58		
Prostration					
No	17/168 (10.1)	Referent		Referent	
Yes	11/29 (37.9)	5.4 (2.2–13.4)	0.0004	5.7 (2.2–14.8)	0.01
Admitted in previous 2 weeks					
No	20/131 (15.3)	Referent			
Yes	2/31 (6.5)	0.4 (0.1–1.7)	0.16		

*Surveillance data are from the Basse Health and Demographic Surveillance System and the Fuladu West Health and Demographic Surveillance System. OR, odds ratio.  †Adjusted for age only. ‡p value for trend. §Defined as weight-for-height z-score <3 SDs from median weight-for-height for the corresponding age group. We calculated weight-for-height using z-scores from the 2006 World Health Organization child growth standards in Stata 14.0 (https://www.stata.com/stata14). Neonates were not included in weight-for-height measurements. ¶The reference ranges for pulse rates were 70–190 beats/min for children <1 month of age, 80–160 beats/min for children 1–11 months of age, 80–130 beats/min for children 1–2 years of age, 80–120 beats/min for children 3–4 years of age, 75–115 beats/min for children 5–6 years of age, 70–110 beats/min for children 7–9 years of age, and 60–100 beats/min for children >10 years of age. #Increased respiratory rate was defined as >60 breaths/min for children <2 months of age, >50 breaths/min for children 2–12 months of age, >40 breaths/ min for children >1–5 years of age.

### Treatment and Susceptibility of Isolates

Among *S. aureus* bacteremia patients identified through referral surveillance, 17.1% (13/76) received initial empiric therapy with cloxacillin, 23.7% (18/76) ampicillin, 31.6% (24/76) penicillin, and 50.0% (38/76) gentamicin; 50 (65.8%) of these patients received >1 antimicrobial drug. The mortality rate did not differ by empiric therapy. Among the 193 *S. aureus* isolates tested, 3.1% were methicillin-resistant (Table 4).

**Table 4:** Antimicrobial drug susceptibility of *Staphylococcus aureus* isolates from children <5 years of age identified through 2 surveillance systems, The Gambia, 2008–2015*

Antimicrobial drug	No. isolates tested	No. (%) sensitive	No. (%) intermediate	No. (%) resistant
Cefoxitin†	193	187 (96.9)	0	6 (3.1)
Chloramphenicol	186	176 (94.6)	2 (1.1)	8 (4.3)
Cotrimoxazole	180	119 (66.1)	21 (11.7)	40 (22.2)
Erythromycin	173	141 (81.5)	24 (13.9)	8 (4.6)
Gentamicin	177	174 (98.3)	0	3 (1.7)
Oxacillin	194	170 (87.6)	24 (12.4)	0
Tetracycline	180	128 (71.1)	2 (1.1)	50 (27.8)

*Surveillance data are from the Basse Health and Demographic Surveillance System and the Fuladu West Health and Demographic Surveillance System. †Cefoxitin was used as a surrogate for methicillin-resistant isolates as recommended by the Clinical and Laboratory Standards Institute (*21*).

## Discussion

We estimated the incidence and CFR of *S. aureus* bacteremia in a rural part of The Gambia using surveillance data over a 5-year period and evaluated trends in incidence over an 8-year period. The incidence was high, particularly among neonates (3.5 cases/1,000 live births), but did not increase with time (Figure 3). The CFR (14.1%) was substantial (Table 1).

The observed incidence of *S. aureus* bacteremia in The Gambia among children 0–59 months of age (78.4 cases/100,000 person-years) was higher than that for industrialized countries (6.5–42.0 cases/100,000 person-years) (*22*,*23*) and some countries of Africa (*4*) and Asia (*24*,*25*), although lower than that reported for Mozambique (*12*). *S. aureus* bacteremia incidence was reported to be 27 cases/100,000 person-years in children <5 years in Kenya (*4*) and 101 cases/100,000 person-years in Mozambique (*12*). In Thailand, a study that reviewed national hospital-based data on bacteremia reported a *S. aureus* bacteremia incidence of 2.5 cases/100,000 person-years (*24*), whereas a population-based study in Bangladesh that focused on children 1–59 months of age with respiratory symptoms reported an incidence of 9.9 cases/100,000 person-years (*25*). The differences in incidence among studies are likely related to the different selection criteria used in the various studies, nutritional status of the patients, presence of concurrent medical conditions, or high levels of antimicrobial drug use without a prescription, especially in Asia (*26*).

During 2008–2015, we found no trend in *S. aureus* bacteremia incidence in The Gambia. Researchers in industrialized countries have shown an increase in the proportion of all bacteremia cases caused by *S. aureus* after the introduction of PCV (*27*). However, our data do not support an association between *S. aureus* bacteremia incidence and the introduction of PCV. Further studies in different settings could help confirm this finding.

The incidence of *S. aureus* bacteremia was highest in neonates, 8 times that reported in Kenya (*4*), and *S. aureus* was the most common cause of bacteremia in this age group. This finding is similar to those of other studies conducted in Africa (*12*,*14*), where *S. aureus* was responsible for 39.0%–56.2% of isolates recovered from neonates. *S. aureus* carriage, which is likely a prerequisite for disease, is also highest during the neonatal period, higher than the carriage of *S. pneumoniae* and *H. influenzae* (*28*). In addition to high rates of acquisition of *S. aureus* during the neonatal period, other reasons for the high risk for *S. aureus* bacteremia might include an immature immune system (*29*).

In our study, only 16% of the *S. aureus* bacteremia cases in neonates presented within the first week of life, unlike for group B *Streptococcus* disease, where 80% of parents seek treatment for their neonates within this period (*30*). Reasons for the difference in timing of treatment might relate to the age at and source of *S. aureus* acquisition.

We found *S. aureus* bacteremia to be more common during the wet season. This seasonal variation might relate to *S. aureus* colonization (a prerequisite for disease), which is highest during the wet season (*31*), or seasonal differences in the incidence of viral infections, which are known to disrupt mucosal epithelium, thereby encouraging *S. aureus* invasion (*32*). In a study of US adults (*33*), the peak incidence of *S. aureus* infection occurred during the winter months and coincided with the peak incidence of viral infections. In Africa, the incidence of viral infections usually peaks during the wet season (*34*), coinciding with the peak *S. aureus* bacteremia incidence.

The CFR in our study was similar to (*10*) or greater than that reported by others (*12*,*23*). Variation in CFRs could be explained by differences between study populations in terms of age, quality of and access to healthcare, focus of infection, antimicrobial drug resistance rates, severity of *S. aureus* bacteremia, or presence of concurrent medical conditions. In our study, 71.4% of deaths occurred on the day of admission, which might be a reflection of severity of disease when care was sought. Even though age is strongly associated with *S. aureus* bacteremia–related death (*10*,*11*), in our study CFR did not vary with age.

The strengths of our study were that the surveillance was population-based and uninterrupted and that blood culture was performed on >84% of eligible patients. However, the study also had some limitations. First, incidence might have been underestimated because some persons with *S. aureus* bacteremia never seek treatment at hospitals and the sensitivity of blood culture is <100%. Second, an increasing rate of enrollment and investigation over time required adjustment of annual case counts. Third, prior use of antimicrobial drugs, although uncommon in our study area, might have reduced the detection of *S. aureus* bacteremia by blood culture. Last, our study was not specifically designed to evaluate risk factors for *S. aureus* bacteremia. For example, *S. aureus* nasal carriage, hospitalization in the previous 6 months, and HIV status were not systematically assessed.

In conclusion, we demonstrated that the incidence of *S. aureus* bacteremia is high in rural Gambia, especially in neonates and infants. Strategies are urgently needed to reduce the burden of *S. aureus* bacteremia and should target children in their first month of life.
